# Auditory Discrimination in Autism Spectrum Disorder

**DOI:** 10.3389/fnins.2021.651209

**Published:** 2021-06-15

**Authors:** Sarah Elizabeth Rotschafer

**Affiliations:** Department of Biomedical Sciences, Mercer University School of Medicine, Savannah, GA, United States

**Keywords:** autism, event-related potentials, sound discrimination, language, cortex

## Abstract

Autism spectrum disorder (ASD) is increasingly common with 1 in 59 children in the United States currently meeting the diagnostic criteria. Altered sensory processing is typical in ASD, with auditory sensitivities being especially common; in particular, people with ASD frequently show heightened sensitivity to environmental sounds and a poor ability to tolerate loud sounds. These sensitivities may contribute to impairments in language comprehension and to a worsened ability to distinguish relevant sounds from background noise. Event-related potential tests have found that individuals with ASD show altered cortical activity to both simple and speech-like sounds, which likely contribute to the observed processing impairments. Our goal in this review is to provide a description of ASD-related changes to the auditory system and how those changes contribute to the impairments seen in sound discrimination, sound-in-noise performance, and language processing. In particular, we emphasize how differences in the degree of cortical activation and in temporal processing may contribute to errors in sound discrimination.

## Introduction

Autism spectrum disorder (ASD) is a developmental condition that is characterized by abnormalities in social communication, restricted behavior, and repetitive behavior. People with ASD also experience altered sensory processing and show both hyper- and hypo-reactivity to sensory input (Heaton et al., 2008; American Psychiatric Association, 2013; Kujala et al., 2013). For reference, between 60 and 96% of people diagnosed with ASD report sensory sensitivities (Schauder and Bennetto, 2016; Kuiper et al., 2019). Responses to auditory stimuli are especially impacted in ASD, with increased sensitivity to noise and difficulty filtering sound from background noise as cardinal features of ASD (Jones et al., 2009; DePape et al., 2012). Behaviorally, individuals with ASD may also be hypersensitive to certain environmental noises, show a decreased tolerance of loud noises, and have a reduced ability to habituate to auditory stimuli (Rosenhall et al., 1999; Khalfa et al., 2004; O’Connor, 2012; Lawson et al., 2015; Bidet-Caulet et al., 2017; Hudac et al., 2018; Ruiz-Martinez et al., 2020). Changes in how sound is received in ASD can also manifest as atypical linguistic processing and comprehension. In addition to delayed language acquisition, individuals with ASD may also show an impaired ability to understand phrases or comprehend single words (Mitchell et al., 2006; Hudry et al., 2010). Ultimately, a significant portion of children with ASD are diagnosed as minimally verbal, and atypical auditory processing may be a contributing factor (Tager-Flusberg et al., 2009; Tager-Flusberg and Kasari, 2013).

These ASD-related changes in auditory behavior and linguistic processing are reflected in dysfunction at the cortical level. Cortical function in ASD is hypothesized to be impacted by an increase in endogenous cortical “noise,” resulting from altered ratios of excitation to inhibition within neural circuits (Rubenstein and Merzenich, 2003; Simmons et al., 2009; Sohal and Rubenstein, 2019). This review therefore will consider the possible causes of ASD-related irregularities in language processing with special attention to how people with ASD process language in natural environmental conditions. As an attempt to understand how impairments in language processing arise in ASD, we will begin by examining errors associated with ASD in processing simple stimuli at the cortical level. In particular, we will focus on the early cortical responses to simple stimuli. Next, we will compare how simple sounds and simple linguistic stimuli are processed, with emphasis placed on cortical responses that track changes in sound stimuli (the mismatch negativity and P300). Lastly, we will review how speech in noise stimuli is represented in ASD. We will discuss aspects of background sounds that make extracting language more difficult in ASD and features that impair linguistic targets’ detection.

## Simple Sounds

A meta-analysis determined that 90% of individuals with ASD experience sensory abnormalities, with auditory hypersensitivity as the most common modality (Gomes et al., 2008). One way in which heightened auditory sensitivity to sound manifests is through pitch differentiation ability as shown through both electrophysiological and behavioral measures. Individuals with ASD perform better than typically developing participants on pitch discrimination tasks, as shown using same-same or same-different testing paradigms (Bonnel et al., 2010). Enhanced pitch discrimination, however, does not necessarily translate into superior linguistic processing ability. Reponses to simple sounds in ASD show atypical sensory peak amplitude and variable peak latencies that may ultimately impair how well individuals with ASD are able to decode language and process environmental noise (Oram Cardy et al., 2008; Port et al., 2016).

To probe cortical responses to sound in ASD, studies generally present participants with various simple sound targets and then measure cortical response using electroencephalograms (EEG) or magnetoencephalograms (MEG). Both EEGs and MEGs represent cortical auditory processing as a series of waveforms (positivities and negativities) that occur in a stereotyped sequential manner as different portions of the cortex become active in response to sound. Generally, EEGs confer greater temporal resolution of responses, while MEGs have superior spatial resolution. Here, we will discuss how “sensory peaks” (P1, N1/M100) respond to simple sounds in ASD, and in how changes in those sounds are represented by the mismatch negativity (MMN).

### P1

P1 is an early cortical response to sound and is thought to reflect thalamocortical transmission along the ascending auditory pathway (Eggermont et al., 1997). In typically developing children, P1 amplitude can track stimulus complexity (amplitude increases with stimulus complexity) (Ceponiene et al., 2001), and can be modulated by arousal (Pratt et al., 2012). Several studies reported reduced P1 amplitude in ASD (Buchwald et al., 1992; Ceponiene et al., 2003a,b; Orekhova et al., 2008; Donkers et al., 2015). Poor early representation of auditory stimuli may subsequently impair the ability of participants with ASD to discriminate between sounds (Ruiz-Martinez et al., 2020). Moreover, in ASD, P1 shows an abnormal lack of modulation in response to changes in the temporal features of sound. In typically developing participants, increasing the presentation rate of a stimulus resulted in attenuation of the P1 amplitude. By contrast, individuals with ASD did not show any change in amplitude as the stimulus presentation rate was modulated, which suggests reduced sensitivity to changes in how sound is represented temporally (Buchwald et al., 1992). Ruiz-Martinez et al. (2020) found a lack of P1 response habituation in ASD to repetitive stimuli, which may indicate a reduced ability to predict and adapt to incoming stimuli, thus contributing to the enhanced auditory sensitivity seen in people with ASD (Table 1).

**TABLE 1:** P1 peak amplitude in response to simple sound stimuli.

**P1 Simple Sounds**	**Research**	**Participants**
Reduced amplitude	Buchwald et al., 1992	Adults with ASD
	Donkers et al., 2015	4–12 year old males with ASD
	Orekhova et al., 2008	4–8 year old males and females with ASD
	Ceponiene et al., 2003b	6–12 year old males with high functioning ASD
	Ruiz-Martinez et al., 2020	5–11 year old males and one female with ASD; included minimally verbal children

*Notable participant features and sub-diagnoses are underlined.*

### N1 and M100

When processing sound, EEGs and MEGs show an event approximately 100 ms after stimulus presentation known as the N1 (EEG) or M100 (MEG). This response is thought to represent activity at the auditory cortex, superior temporal gyrus, and auditory association areas. There are also data suggesting parietal and frontal cortex involvement (Naatanen and Picton, 1987). In typically developing people, the N1/M100 latency is longer in children and shortens as they age. In children with ASD however, M100 latencies are more variable. With regard to auditory response development, peak latencies in children with ASD were reported to change with age in a manner similar to results seen in typically developing children in the left hemisphere of the brain, but M100 latencies in the right hemisphere did not change with age (Gage et al., 2003b). Other studies cataloged unusually long N1/M100 latencies in a variety of testing paradigms (Oades et al., 1988; Bruneau et al., 1999; Seri et al., 1999; Korpilahti et al., 2007; Sokhadze et al., 2009; Roberts et al., 2010; Port et al., 2016; Table 2). Port et al. (2016) tested children with ASD once when they were between the ages of 6 and 11 years, then again 5 years later. They had participants passively listen to a series of pure tones and found delays in the M100 response latency at both time points and discovered a relationship between M100 delay and clinical ASD severity. Seri et al. (1999) specifically tested children with tuberous sclerosis and found delayed peak latency, showing that at least for tuberous sclerosis, the results from a specific sub-diagnosis of ASD were consistent with ASD results on the whole. Similarly, longer N1 latency was present in children with Asperger syndrome (Korpilahti et al., 2007). Oades et al.’s (1988) approach was slightly different from the other listed studies in that they asked children with ASD to perform a task while listening to test stimuli—the participants were instructed to press a button when they heard a target tone stimulus and ignore non-target distractor tones. Interestingly, Oades et al. (1988) found that N1 latencies were longer in response to non-target stimuli but shorter in response to target stimuli. It is possible then that variation in N1 latencies may be a correlate of abnormalities in how children with ASD direct their attention when performing auditory tasks (Oades et al., 1988). Ferri et al. (2003) reported shorter peak latencies in children with ASD, though they noted that their study used children diagnosed with ASD and intellectual impairment as participants, while similar research tended to base their results on high functioning children with ASD. As such, they raise the possibility that the degree of intellectual impairment present in children with ASD may impact response latencies (Ferri et al., 2003). However, Bruneau et al. (2003) also recruited children with ASD and intellectual impairment but found the opposite result—longer peak latencies. As a point of differentiation, Bruneau et al. (2003) and Ferri et al. (2003) used distinctly different age groups, meaning that multiple factors may act in combination to influence auditory responsiveness in ASD.

**TABLE 2:** N1 peak in response to simple sound stimuli.

**N1 simple sounds**	**Research**	**Participants**
Greater Amplitude	Flagg et al., 2005*	8–17 year old males with ASD
	Gage et al., 2003a*	8–14 year old males with ASD
	Dawson et al., 1986*	6–18 year old males with ASD; some with intellectual impairment
	Rojas et al., 2001	Adults with fragile X syndrome
	Van der Molen et al., 2012a,b	18–42 year old males with fragile X syndrome
	Castren et al., 2003	7–13 year old males with fragile X syndrome
Longer Latency	Port et al., 2016	Mean age 8 years old at initial recruitment; males
	Roberts et al., 2010*	Mean age 10 years old; sex not reported
	Sokhadze et al., 2009*	9–27 year old males and one female with high functioning ASD
	Bruneau et al., 1999*	4–8 year old males and females with intellectual impairment and ASD
	Seri et al., 1999	7–10 year old with tuberous sclerosis; sex not reported
	Korpilahti et al., 2007	9–12 year old males with Asperger syndrome
	Oram Cardy et al., 2008*	7–18 year old males and females with ASD and/or Asperger syndrome
Shorter/Atypical Latency	Gage et al., 2003b	8–14 year old males with ASD
	Oades et al., 1988	6–18 year old males and one female
	Ferri et al., 2003	6–19 year old males with ASD and intellectual impairment

*Specific sub-diagnoses and notable features of participants are underlined. Asterisks indicate that the reported result was only seen in the right hemisphere.*

Work examining N1/M100 in people with ASD frequently reported abnormalities in lateralization. In typically developing people, the left temporal cortex response to sound is generally greater than that of the right temporal cortex (Eyler et al., 2012). However, several studies that included participants with ASD reported prolonged latencies specific to the right hemisphere (Table 2; Bruneau et al., 2003; Gage et al., 2003b; Oram Cardy et al., 2008; Sokhadze et al., 2009; Roberts et al., 2010), and an overall increase in right hemisphere responsiveness to sound (Dawson et al., 1986; Gage et al., 2003a,b; Flagg et al., 2005). These findings correlate well with MRI work showing superior temporal gyrus activity to be symmetrical (as opposed to showing a leftward bias) in adults with ASD as a result of increased right hemisphere superior temporal gyrus volume (Jou et al., 2010). Prolonged right hemisphere latencies are consistent with results showing developmental delays in ASD and suggest that some of the errors seen ASD in sound processing may stem from abnormalities in gross neuroanatomy.

As a general statement, people with ASD seem to show greater peak amplitudes and longer peak latencies for N1/M100. This trend is informative with regard to how auditory function may be fundamentally altered in ASD at early stages of cortical processing. Delays in peak latency correlate with stimulus complexity and the recruitment of neural resources. In which case, longer peak latencies and broader recruitment of neural resources could mean that individuals with ASD find simple stimuli to be more complex than control participants generally do (Lepisto et al., 2008). These findings may also be a byproduct of a loss of long-range connections in the ASD brain, causing people with ASD to rely more heavily on local connections to process sound stimuli (Jou et al., 2010).

### MMN and MMF

The mismatch negativity is a waveform that reflects changes in stimuli; generally, it is considered to act as an automatic orienting reflex that marks changes in an environment. The MMN can be modulated by participants focusing their attention on a stimulus, but can still be elicited when attention is not directed at stimuli (Alho, 1995; Naatanen and Alho, 1995). The MMN is thought to represent activity at the auditory cortex that has been supplemented by inputs from the frontal lobe. It may also reflect activity at the hippocampus and thalamus (Alho, 1995; Garrido et al., 2009). In studies using magnetoencephalography, this waveform is referred to as the magnetic mismatch field latency (MMF). Because MMN/MMF tracks changes in stimuli, it is often studied using some variation of an “oddball” task, where participants listen to a stream of identical standard sounds that have a target stimulus (a stimulus that deviates from the standard sounds in some metric) or novel stimulus interleaved.

Research that tested the MMN amplitude in response to pure tone stimuli in ASD reported a range of findings that may reflect variation in participants’ sub-diagnosis, degree of intellectual impairment, tolerance of change, or age (Gomot et al., 2002, 2011; Ceponiene et al., 2003b; Ferri et al., 2003; Tecchio et al., 2003; Vlaskamp et al., 2017; Table 3). Ferri et al. (2003) and Gomot et al. (2011) found that children with ASD and intellectual impairments had larger MMN responses to deviant stimuli, although a MEG study that also focused on low functioning individuals with ASD found reduced MMF amplitude (Tecchio et al., 2003). While both Ferri et al. (2003) and Tecchio et al. (2003) surveyed people with ASD and intellectual impairment, Ferri et al. (2003) put more focus on younger individuals (6–19 years) and excluded participants with known chromosomal abnormalities (fragile X syndrome, tuberous sclerosis, etc.). Tecchio et al. (2003) drew participants from a greater range of ages (8–32 years) and did not seek to exclude certain sub-diagnoses. Gomot et al. (2011) also saw heighted MMN responses in ASD and found that MMN amplitude was associated with participants’ ability to tolerate change. As such, the variability in MMN amplitude results may reflect age-related differences and/or differences in how certain sensory impairments manifest in ASD. Consistent with this line of thought, work that tested children with high functioning children ASD generally did not see any significant MMN differences, suggesting that the sub-diagnosis and the degree of intellectual impairment of participants could significantly impact the MMN profile (Gomot et al., 2002; Ceponiene et al., 2003b). Notably, when specific conditions on the autism spectrum were considered, the results were uneven. MMN peak amplitude was reduced in fragile X syndrome (Van der Molen et al., 2012b), and abnormally prolonged Rett syndrome (Stauder et al., 2006; Foxe et al., 2016), lending credibility to the notion that subtle variations sub-diagnosis phenotype may drive some of the inconsistencies found in MMN results. Vlaskamp et al. (2017) also sought to explain some of the variability in ASD MMN findings by testing the ASD response to frequency and duration deviants in an oddball task using a relatively large number of participants in a more discrete age range (8–12 years). That work found that MMN amplitude was reduced in individuals with ASD to both frequency and duration deviants (Vlaskamp et al., 2017), which may reflect a lessened ability to track certain types of changes in auditory stimuli in ASD.

**TABLE 3:** MMN peak amplitude and latency in response to simple sound stimuli.

**MMN simple sounds**	**Research**	**Participants**
Greater Amplitude	Gomot et al., 2011	5–11 year old males and females
	Ferri et al., 2003	6–19 year old males with ASD and intellectual impairment
No difference in Amplitude	Gomot et al., 2002	5–9 year old males and females with ASD
	Ceponiene et al., 2003b	6–12 year old males with high functioning ASD
Abnormal waveform	Foxe et al., 2016	4–21 year old girls with Rett syndrome
Reduced Amplitude	Tecchio et al., 2003	8–32 year olds; ASD and intellectual impairment
	Vlaskamp et al., 2017	8–12 year old males and females with ASD and/or Asperger syndrome
	Van der Molen et al., 2012a	18–42 year old males with fragile X syndrome
Longer Latency	Matsuzaki et al., 2017	Mean age 9.5 year old males with ASD and auditory sensitivity
	Jansson-Verkasalo et al., 2005	Mean age 11 years old; males with Asperger syndrome
	Seri et al., 1999	7–10 year old with tuberous sclerosis; sex not reported
	Foxe et al., 2016	4–21 year old girls with Rett syndrome
Shorter latency	Gomot et al., 2002	5–9 year old males and females with ASD

*Notable participant features and sub-diagnoses are underlined.*

Similar to research describing MMN/MMF peak amplitude, data describing MMN/MMF latency vary by the study design and the participant composition. Consistent with the superior pitch discrimination observed in ASD, Gomot et al. (2002) found that MMN latency was shorter in children with ASD. There was also evidence suggesting that the MMN response in ASD may receive contributions from generators other than those used in typically developing controls, which may partially explain the observed change in MMN latency (Gomot et al., 2002). By contrast, other work that specifically examined the relationship between auditory sensitivity (as determined by performing a sensory profile assessment) and MMF latency found that participants who had ASD and atypical auditory sensitivity also tended to have prolonged MMF latencies (Matsuzaki et al., 2017). Studies that tested children with ASD using pure tones in an oddball paradigm also found longer MMN/MMF latencies (Seri et al., 1999; Jansson-Verkasalo et al., 2005; Oram Cardy et al., 2005b; Matsuzaki et al., 2017). Seri et al. (1999) reported longer MMN latencies in children diagnosed with ASD and tuberous sclerosis. Similarly, research studying Asperger syndrome, a condition that has subsequently been categorized as ASD by the DSM-5, also found delayed MMN responses (Jansson-Verkasalo et al., 2005). These instances then provide additional support for the idea that variability in processing simple sounds may be tied to specific impairments in sub-diagnoses and/or auditory sensitivity (Table 3). Moreover, changes in MMN latency track task difficulty. Therefore, increased peak latencies could also be an artifact of participants with ASD finding auditory tasks more difficult than did typically developing participants (Garrido et al., 2009).

## Speech Sounds

### Vowels and Phonemes

People with ASD demonstrate abnormal responses to phonemes and other speech-like sounds, which may stem from an altered perception of less complex stimuli or from impairments in auditory attention that are specific to linguistic components. Phonemes are the units of sound that comprise a language. Therefore, while phonemes have simple and complex tonal components, they have additional meaning in that they are used in an inherently social context. Aberrations in how “simple” stimuli are processed may hinder people with ASD in processing phonemes to some extent; however, there is also evidence that processing difficulties may be unique to linguistic elements.

### MMN and MMF

The MMN response to phonemes is somewhat varied, though some trends have emerged. Some studies reported that MMN/MMF peaks from participants with ASD were not different from those seen in typically developing children when presented with vowel sounds (Kemner et al., 1995; Ceponiene et al., 2003b; O’Brien et al., 2020). However, Kuhl et al. (2005) studied low-functioning pre-school aged children using a speech versus computer synthesized non-speech oddball task and found that although the MMN generated in response to non-speech sounds was intact, the MMN peak was lost in children with ASD in response to syllable changes (Table 4) (Kuhl et al., 2005). Interestingly, an fMRI study that examined voice active portions of the brain (superior temporal sulcus) found a similar result—participants with ASD showed a lack of activity when they were presented with vocal sounds, but showed no difference from controls when listening to non-vocal stimuli (Gervais et al., 2004).

**TABLE 4:** MMN in response to vowel and phoneme stimuli.

**MMN vowels and phonemes**	**Research**	**Test**	**Participants**
Longer latency	Kasai et al., 2005	Phoneme changes	Mean age 27 year old males and females with ASD
	Matsuzaki et al., 2019	Oddball using vowels	Mean age 22 year old males with ASD
	Galilee et al., 2017	Speech vs non-speech	4–6 year old males and females with high functioning ASD
	Roberts et al., 2011	Vowel vs tone oddball	6–15 year old males and females with ASD (Asperger syndrome included) and language impairment
No latency or amplitude differences	Ceponiene et al., 2003b	Vowel sounds	6–12 year old males with high functioning ASD
	Kemner et al., 1995	Vowel sound oddball	7–13 year old males and female; ASD with intellectual impairment
	O’Brien et al., 2020	Vowel and tone oddball	5–15 year old males and females with tuberous sclerosis
Greater amplitude	Lepisto et al., 2008	Pitch or phoneme-type changes in speech stimuli	7–11 year old boys with ASD
	Lepisto et al., 2005	Pitch, duration, and vowel changes in speech and non-speech stimuli oddball	7–11 year old males with ASD; lower verbal IQ in ASD group
	Lepisto et al., 2006	Pitch, duration, and vowel changes in speech and non-speech stimuli oddball	8–11 year old boys with Asperger syndrome
Reduced amplitude	Kuhl et al., 2005	Speech vs computer synthesized non-speech oddball task	3–4 year old males and females with ASD; low functioning

*Specific sub-diagnoses and notable features of participants are underlined.*

Magnetic mismatch field latency was generally delayed in vowel discrimination oddball tasks for individuals with ASD (Oram Cardy et al., 2005b; Roberts et al., 2011; Matsuzaki et al., 2019). When tested with across-phoneme changes (switching between the vowels /a/ and /o/), individuals with ASD showed prolonged MMF latencies over the left hemisphere which were correlated with symptom severity. Varying pure tone or vowel duration, however, did not reveal any ASD-related differences (Kasai et al., 2005). Therefore, while changing the physical aspects of stimuli did not seem to elicit changes in MMN, rapid switching between vowel stimuli did, which may mean that the difficulty that people with ASD experience in processing language may be partially rooted in a poor ability to follow temporal cues in a linguistic context (Kasai et al., 2005).

Lepisto et al. (2005, 2006) designed a task that introduced pitch, duration, and vowel changes to speech and non-speech stimuli and tested children with ASD and Asperger syndrome. Results showed MMN enhancement in response to speech and non-speech pitch changes in both groups (Lepisto et al., 2005, 2006). To elaborate on these results, Lepisto et al. (2008) tested for differences in MMN peaks elicited by changes in pitch or phoneme-type in speech stimuli. To that end, they created a paradigm where either pitch stimuli or phoneme stimuli were presented, and either (1) the features of the standard and deviant stimuli were unaltered, or where (2) irrelevant variations were introduced to the standard and deviant stimuli (stimuli were varied with regard to pitch in the phoneme deviant category or with regard to phoneme in the pitch deviant category). Children with ASD had elevated MMN amplitude in response to pitch changes in both conditions, and for phoneme changes in the first condition. However, the MMN enhancement seen in response to phoneme changes in the ASD group was lost in the second, more speech-like condition (Lepisto et al., 2008). The authors suggest that the enhanced simple sound processing typical of children with ASD may make processing linguistic stimuli more difficult because they may be less able to ignore irrelevant cues (Lepisto et al., 2008; Cheng et al., 2017). Or, put another way, in the context of speech, people with ASD may be too overly focused on “low level” acoustic cue variation to track phoneme changes (Lepisto et al., 2008; Cheng et al., 2017).

In summary, work that studied the ASD MMN/MMF in response to speech stimuli varied with intellectual impairment, ASD subtype, and the nature of the auditory task. MMN did not seem to be especially sensitive to changes in the physical features of simple sounds (pitch and duration); however, it was markedly altered in response to vowel changes. This may imply (1) that there are stimulus features specific to language that slow auditory processing and (2) that impaired ability to rapidly detect changes in incoming speech stimuli may be fundamental to ASD language processing deficits. Consistent with this line of thought, Lepisto et al. (2005, 2006, 2008) saw enhanced MMNs in response to changes in speech stimuli suggesting that individuals with ASD devote more processing power to encoding low level features of linguistic stimuli.

### P3

The P3a is a response to unexpected or surprising stimuli and is thought to reflect activity in the frontal, temporal, and parietal lobes (Polich, 2007). As such, it is often tested using an oddball paradigm. The P3a in particular is associated with the detection of novelty and the ability to orient to a stimulus (Yamaguchi and Knight, 1991; Verleger et al., 1994). This is significant as children with ASD routinely show impaired sound orientation for both social and non-social stimuli (Dawson et al., 1989).

In two different studies, reduced P300 amplitude was seen in response to phonemes but not to tonal stimulus. In the first, Dawson et al. (1988) presented a phonetic “da” and a piano chord as stimuli to children with ASD (Table 5). While there was no apparent change in how the piano chord was processed between groups, the children with ASD showed a significant reduction in P3 size in the left hemisphere in response to the “da” phoneme (Dawson et al., 1988). This finding was consistent with later work showing P3a reductions in ASD participants on oddball tasks that used speech stimuli (Kemner et al., 1995; Lepisto et al., 2005, 2006), and work showing reduced amplitude in fragile X syndrome, a condition on the autism spectrum (St Clair et al., 1987; Van der Molen et al., 2012a,b). In the second study, Ceponiene et al. (2003b) had high functioning children with ASD undergo an oddball task using simple tones, complex tones, and vowel sounds as stimuli. They found that there were no differences in the P3a response elicited by simple and complex stimuli between the ASD and typically developing groups, but when presented with vowels, the P3a disappeared in the ASD group (Ceponiene et al., 2003b). Comparable results were also found in individuals with Rett syndrome, a condition on the autism spectrum (Stauder et al., 2006). Taken together, these results indicate that vowel and phoneme processing is uniquely impacted in ASD and specific genetic conditions on the autism spectrum.

**TABLE 5:** P3 latency and amplitude in response to vowel and phoneme stimuli research.

**P3 vowels and phonemes**	**Research**	**Test**	**Participants**
Missing	Ceponiene et al., 2003b	Oddball with simples tones, complex tones, and vowels	High functioning ASD
	Stauder et al., 2006	Audiovisual oddball	2–60 year old females with Rett syndrome
Reduced amplitude	Dawson et al., 1988*	Phoneme vs piano cord	6–18 year old males with ASD; some with intellectual impairment
	Kemner et al., 1995	Vowel sound oddball	7–13 year old males and female with ASD and intellectual impairment
	Lepisto et al., 2005	Pitch, duration, and vowel changes in speech and non-speech stimuli oddball	7–11 year old males with ASD; verbal IQ lower in ASD group
	Whitehouse and Bishop, 2008	Vowel and complex sound oddball	8–14 year old males with ASD; verbal IQ lower in ASD group
	Lepisto et al., 2006	Pitch, duration, and vowel changes in speech and non-speech stimuli oddball	8–11 year old boys with Asperger syndrome
	Van der Molen et al., 2012a,b	Auditory tonal stimuli	Mean age 29 years old; males with fragile X syndrome
	St Clair et al., 1987	Oddball task	Adults with fragile X syndrome

*Notable features of participants and sub-diagnoses are underlined. Asterisk indicates results only applied to phoneme stimuli.*

Whitehouse and Bishop (2008) expanded on these findings by testing the role of attention in speech sound processing. They saw a general reduction in peak amplitudes when children with ASD passively listened to speech sounds, but peak amplitudes were restored when they were required to actively attend to the speech stimuli (Whitehouse and Bishop, 2008). Interestingly, they also found that children with ASD were less likely to orient to novel tones that were embedded in a stream of speech sounds, but orienting was intact when speech sounds were embedded in tonal stimuli. These results show that, first, responses to speech can be modulated by attention in ASD, and second that participants with ASD are able to attend to speech stimuli depending on the context in which speech sounds are presented. The second finding runs somewhat contrary to work showing reduced orienting to speech in children with ASD (i.e., Dawson et al., 1998; Ceponiene et al., 2003b; Kuhl et al., 2005) in that orienting to speech was possible in ASD under specific conditions.

## Speech in Noise

Research that studied how people with ASD process phonemes focused on how specific components of speech may impede processing in ASD. Those inquiries found that people with ASD had difficulty following phoneme changes, may become distracted by contextually irrelevant features of language, and may show attentional deficits with regard to linguistic stimuli. In addition to the challenges that seem to be intrinsic to speech processing in ASD, individuals with ASD experience particular difficulty processing sound when it is presented amid environmental noise. Background noise can impact simple sound detection, but can also impair linguistic processing to a greater or lesser degree depending on the features of the background noise stimuli. The presence of background stimuli could also contribute to the heightened cortical “noise” that is thought to interfere with auditory function in ASD (Rubenstein and Merzenich, 2003; Sohal and Rubenstein, 2019).

### Simple Sounds and Phonemes in Noise in ASD

Even relatively simple background noise can increase the processing load in ASD to the point at which target identification is impaired. To investigate how the presence of background noise affected simple sound processing in ASD, Mamashli et al. (2017) probed the neural generators of MMF using pure tones against either a quiet or a multi-speaker babble background. In the quiet condition, no difference was found in MMF between groups; however, activation of the inferior frontal gyrus, a generator of MMF, was reduced in the noise condition. Because the inferior frontal gyrus acts to evaluate syntax in incoming language (Tanaka et al., 2017), reduced inferior frontal gyrus activation is consistent with a processing impairment that is specific to language. Moreover, in examining how MMF generators coordinate activity, measures of functional connectivity revealed increased recruitment of neural resources in ASD for both the quiet and the noisy conditions (Mamashli et al., 2017). This suggests that the impact of background noise on speech perception may be a by-product of a general over-responsiveness to sound in ASD.

In this vein, Russo et al. (2009) tested cortical responses to phonemes in children with ASD in noisy conditions. Children either listened to the syllable /da/ with a silent background or while embedded in a white noise background. As might be expected, typically developing children tended to show delayed and reduced responses to stimuli presented in the background noise condition. The ASD group, however, showed only a very slight difference between their responses to the quiet and noisy background conditions. This implies that children with ASD experience a more involved cortical response when processing speech stimuli regardless of the background (Russo et al., 2009). As such, the additional demands of performing more complex speech discrimination tasks (such as identifying words and sentences) against a noisy background may exacerbate problems in what are already tenuous language processing skills.

### Speech in Noise Detection in ASD

While background noise impairs speech detection in ASD generally, the features of the background noise in which those speech targets are presented can also impact how well participants are able to extract speech. For instance, people with ASD were found to perform poorly in speech discrimination tasks when the background noise contained temporal dips. Most often, tests aimed at determining how well individuals with ASD are able to extract speech sounds from background noise consist of having participants identify either a word or portions of a sentence while simultaneously presenting a background noise that competes with the target in some way. To test how spectral and temporal variations in background noise effected individuals with ASD’s ability to detect speech, Groen et al. (2009) designed an experiment where participants were instructed to repeat back target words presented against a “pink noise” background (noise with spectral energy divided evenly across the frequency bands of the human auditory system). To specifically test the effect of temporal variation, Groen et al. (2009) created pink noise backgrounds that either had or did not have temporal dips. Performance in both the typically developing and ASD group suffered when temporal dips were introduced, but the degree of disruption was greater in the ASD group (Groen et al., 2009). Similarly, when asked to extract speech (whole sentences) from a sampling of different background noise conditions, the performance of participants with ASD was significantly worse in conditions where temporal or spectro-temporal dips were introduced into a speech-like background (Alcantara et al., 2004). It is hypothesized that during temporal dips in background noise, typically developing listeners can piece together the target speech using contextual cues, but listeners with ASD are less able to gather or use those cues (Alcantara et al., 2004; Qian and Lipkin, 2011). In addition, work that focused on speech detection against an “attention demanding” multi-talker background stimulus found that individuals with ASD performed significantly worse than their typically developing counterparts (Dunlop et al., 2016). Taken together, these results indicate that individuals with ASD may be less able to integrate information gathered during breaks in background stimuli, and, as difficulty extracting target speech against multi-talker background suggests, individuals with ASD may also require a greater signal-to-noise ratio to discriminate spoken words.

Schelinski and von Kriegstein (2020) tested whether signal-to-noise ratio differences in ASD were a driving factor for speech in background noise discernment errors. In their experiment, typically developing adults and adults with ASD listened to speech presented against a continuous speech-shaped background noise. Results showed that typically developing participants could detect target stimuli at a significantly lower sound-in-noise ratio than the participants with ASD. Put another way, the difference in intensity between the signal and background noise had to be greater for the group with ASD to detect the target signal. These results are somewhat contrary to those of Alcantara et al. (2004) and Groen et al. (2009) in that they show target detection impairment in ASD with a continuous noise background rather than only in temporally shaped background noise. However, in the interest of allowing for greater expression of ASD symptom variability, Schelinski and von Kriegstein (2020) did use less challenging speech recognition thresholds in their research than those used in previous studies. This may mean that temporal processing deficits in ASD could be ameliorated by conditions with a more favorable signal-to-noise ratio (Schelinski and von Kriegstein, 2020).

As another possibility, individuals with ASD may find speech in noise conditions challenging as a result of altered voice perception. Schelinski and von Kriegstein (2020) also found that speech-in-noise recognition correlated with vocal pitch perception ability in typically developing adults, but not in adults with ASD. This effect seems to be limited to vocal pitch discrimination however, as testing vocal timbre discrimination did not reveal differences between typically developing and ASD groups (Schelinski et al., 2017). In keeping with the idea that poor voice recognition contributes to poor sound in noise performance, fMRI data also demonstrated that participants with ASD had an impaired ability to recognize a target speaker’s voice as shown by reduced activity in the superior temporal sulcus and superior temporal gyrus (temporal voice areas) (Schelinski et al., 2014, 2016).

While individuals with ASD do seem to experience aberrations specific to processing speech and voices, given that speech is an inherently social activity, the desire to engage in social exchange must also be considered when testing speech-in-noise processing. When participants with ASD were presented with a conversation between two people that had competing “ecologically valid” background noise (noise typical of everyday social situations), typically developing and participants with ASD had comparable patterns of brain activity (Hernandez et al., 2020). However, the angular gyrus was relatively more active in participants with ASD and angular gyrus activity was correlated with social motivation (as measured by the Social Responsiveness Scale—2nd Edition, SRS-2). Therefore, Hernandez et al. (2020) speculate that participants with higher social motivation would be more likely to direct attention to the conversational sound in noise targets, and therefore may perform better than their less socially motivated peers in recalling conversational targets (Hernandez et al., 2020).

## Discussion

### Developmental Delays and Simple Stimuli Processing in ASD

Delayed or atypical development of sensory systems is a common feature of ASD and may underlie the peak latency and amplitude changes observed in EEG/MEG testing. In typically developing individuals, waveforms become more complex as children age. Importantly, auditory development in early childhood sees reductions in P1 latency and amplitude as N1 becomes more prominent (Oades et al., 1997; Sharma et al., 1997; Ponton et al., 2000; Wunderlich et al., 2006). Early developmental changes also track reductions in N1 latency (Bruneau et al., 1997; Oades et al., 1997; Sharma et al., 1997; Ponton et al., 2000; McArthur and Bishop, 2002). Several of the studies reviewed here reported significant ASD-related delays in N1 latency results which could reflect under-development of the auditory system. Consistent with this finding, work that compared peak latency with participant’s age failed to find any age-related change in M100 peak latency over the right hemisphere (Gage et al., 2003a), reinforcing the idea that the errors found at N1 may be developmental in nature.

The majority of studies that characterized N1 also found increases in peak latency and amplitude that were almost exclusively seen over the right hemisphere, suggesting poor response lateralization in ASD. In typically developing people, auditory areas of the brain (including the STG) undergo synaptic pruning events that cause decreases in size relative to brain volume between childhood and young adulthood. During this developmental period, a left-right hemisphere asymmetry in auditory area volume is established. Symmetry in auditory area size in ASD seems to be rooted in a failure to loose volume in the right (or non-dominant) hemisphere of the brain (Devous et al., 2006; Jou et al., 2010). As such, the larger responses seen over the right hemisphere in ASD may simply reflect larger right hemisphere EEG/MEG generators that are a consequence of impaired synaptic pruning during development.

With regard to P1, peak amplitude was reduced in ASD in all studies reviewed, including studies with participants as young as 4 years old. This shows that even from a young age, sensory processing is abnormal in children with ASD (Orekhova et al., 2008; Donkers et al., 2015). P1 also seemed insensitive to changes in the timing of stimuli and lacked a habituation response in ASD (Buchwald et al., 1992; Ruiz-Martinez et al., 2020); as such, changes in stimuli was not well represented by P1. Functionally, the inability to track changes in a stimulus may manifest as poor sensory gating in ASD. Similarly, a lack of habituation to stimuli could conceivably contribute to the heightened auditory sensitivity typical of ASD. Impaired representation of stimulus timing may ultimately play a role in the linguistic deficiencies found in ASD, as individual with ASD may have difficulty following spectro-temporal changes in words.

### Arousal and Attention in ASD

At rest, individuals with ASD show differences in their states of arousal (as measured by skin conductance, body temperature, and heart rate) relative to control groups (Schoen et al., 2009; Mathersul et al., 2013; Eilam-Stock et al., 2014; Prince et al., 2017). Participants’ state of arousal is relevant to auditory testing because arousal impacts the size of the EEG/MEG peaks observed. Kozlowska et al. (2017) found that an individual’s baseline state of arousal seems to act as a precondition to response magnitude. When tested with an auditory oddball task, children with neurologic disturbances that caused heightened arousal showed greater amplitudes in ERP components (Kozlowska et al., 2017). Additionally, children with higher arousal (as measured by heart rate) tended to show larger P3a peaks on an oddball task (Wass et al., 2019). Taken together, it is possible that changes in ASD participants’ state of arousal during testing may drive some of the variability found in ASD auditory EEG/MEG data; therefore, future studies may consider including a control for arousal when testing auditory processing in ASD.

Differences in how people with ASD direct their attention when undergoing auditory EEG/MEG testing could also account for some of the observed inconsistencies in the literature. Individuals with ASD consistently show anomalies in joint attention and orienting to speech, and frequently show co-morbidity with attention deficit/hyperactivity disorder (reviewed in Mundy, 2018; Sharma et al., 2018). Auditory attention sharpens frequency tuning and can act to enhance gain for target stimuli (de Boer and Krumbholz, 2018). It would follow then that ASD-related anomalies in how attention is directed could drive atypical results in simple sound testing, as seen in Oades et al. (1988). Measures of attention have also been used to predict speech in noise ability (Moore et al., 2010), which is consistent with the attentional effects described in studies that asked participants with ASD to extract speech sounds from background noise (Whitehouse and Bishop, 2008; Dunlop et al., 2016; Hernandez et al., 2020).

### ASD Sub-Diagnosis in Auditory Processing

Idiopathic ASD is the most common form of ASD; however, that diagnosis may describe a number of genetic conditions that potentially have slightly different presentations. Comparing non-syndromic ASD with known genetic conditions on the autism spectrum is useful in interpreting some of the variability found in ASD EEG/MEG data. For instance, Rett syndrome is a condition on the autism spectrum that presents with cognitive impairments. Consistent with ASD results, individuals with Rett syndrome show general increases in early sensory peak latencies (Stauder et al., 2006; Foxe et al., 2016), but divergent MMN results. Also, while the majority of ASD studies reported reduced P3 amplitudes, P3 was missing entirely in Rett syndrome (Stauder et al., 2006). Tuberous sclerosis, another autism spectrum condition, is characterized by unchecked protein synthesis and tumor growth that cause abnormal neural connections, longer peak latencies, and asymmetrical N1 response lateralization (Seri et al., 1999; Feliciano et al., 2013; O’Brien et al., 2020). Excess protein production is also found in fragile X syndrome, which renders EEG/MEG results that are mostly in line with typical ASD responses—N1 amplitude is greater, latency is longer, and P3 waveforms are abnormal (St Clair et al., 1987; Rojas et al., 2001; Van der Molen et al., 2012a,b). Unlike Rett syndrome patients however, participants with fragile X show ASD-like N1 lateralization (Rojas et al., 2001). Taken as a whole, fragile X syndrome and Rett syndrome data show that even conditions with similar etiologies can produce subtly different EEG/MEG results.

### Temporal Integration in ASD

The capacity to distinguish speech sounds and parse speech depends on the ability to follow rapid temporal cues on the order of milliseconds. As such, it is conceivable that even small changes in temporal processing may ultimately have a significant impact on an individuals’ ability to perceive and understand speech (Tallal et al., 1993). Gap detection tests measure auditory temporal processing ability by presenting listeners with a series of sounds and varying the interval of time between presentations. The goal of these tests is to determine the interval duration at which listeners are able to perceive the sounds as discrete stimuli rather than as a single continuous sound. Gap detection testing has consistently shown that children with ASD tend to need longer gaps in order to identify stimuli, which suggests that children with ASD experience impaired temporal resolution in processing sound (Kwakye et al., 2011; Bhatara et al., 2013; Foss-Feig et al., 2017). Recent literature also found that not only did children with ASD require longer gaps to parse sounds, but that gap detection ability was correlated with lessened phonological awareness and impaired speech-in-noise detection (Foss-Feig et al., 2017). Similarly, MEG work showed children with ASD failed to respond to the second stimulus when duos of pure tones were presented in rapid succession, supporting the idea that rapid temporal processing is impaired in ASD (Oram Cardy et al., 2005a). In point of fact, all EEG/MEG peaks but P1 are sensitive to changes in stimulus presentation rate (Dinces and Sussman, 2011). This suggests that at least some of the difficulty that children with ASD have in attending to syllables/words/sentences arise from temporal processing impairments (Bhatara et al., 2013; Foss-Feig et al., 2017).

## Further Considerations

In review, some consistent themes emerged with regard to ASD EEG/MEG data, but still many EEG/MEG components reported a range of responses. This is at least somewhat expected given the variety of sub-diagnoses, intellectual ability, and developmental delays represented under to ASD umbrella. In order to better understand the driving factors in ASD-related sensory disability, future studies may incorporate the following five considerations. First, studies that required participants to direct their attention toward or away from a stimulus found consistent changes in EEG/MEG responses (de Boer and Krumbholz, 2018). Given that people with ASD routinely exhibit difficulties around attentional focus, incorporating an assessment of attentional ability may be worthwhile. Second, because state of arousal does have an impact on EEG/MEG response magnitude, monitoring participants’ state of arousal during testing may also aid in understanding abnormal responses. Third, some studies made efforts to control for developmental delays by using not only age matched, but developmentally aged matched controls for participants with ASD. Such endeavors can be beneficial in interpreting aberrant data, but enforcing age range restrictions of participants included in a study may decrease ambiguity in the results. Fourth, the ASD umbrella represents a wide range of both known and yet-to-be-identified genetic conditions. Given that known genetic conditions give varying EEG/MEG results, it is not unreasonable to suppose that the conditions represented in idiopathic ASD may also provide idiosyncratic results. As such, including any efforts that have been made to identify the underlying cause of participants’ ASD in reports would be beneficial. Lastly, the majority of the literature studying auditory processing in individuals with ASD used exclusively male participants. While ASD does seem to be more common in males, ASD is also thought to be underdiagnosed in females. In order to better understand and diagnose ASD in girls, a complete picture of the sensory issues they face is essential.

## Author Contributions

The author researched and wrote the entire manuscript.

## Conflict of Interest

The author declares that the research was conducted in the absence of any commercial or financial relationships that could be construed as a potential conflict of interest.
